# Impact of Germline DNA Repair Mutations on Clonal Hematopoiesis and Myeloid Neoplasm Development

**DOI:** 10.1007/s11899-025-00768-9

**Published:** 2025-12-29

**Authors:** Kateryna Fedorov, Leo Y. Luo, Alexander G. Bick, Michael R. Savona

**Affiliations:** 1 Vanderbilt-Ingram Cancer Center, TN Nashville, US; 2 Division of Hematology and Oncology, Department of Medicine, Nashville, TN US; 3 Department of Radiation Oncology, Nashville, TN US; 4 Division of Genetic Medicine, Department of Medicine, Nashville, TN US; 5 Vanderbilt School of Medicine, Vanderbilt University Medical Center, Nashville, TN US

**Keywords:** Clonal hematopoiesis, DNA Damage Repair mutations, Germline predisposition, Myeloid Neoplasms

## Abstract

**Purpose of Review:**

Clonal hematopoiesis (CH) arises from the expansion of a single hematopoietic stem cell harboring somatic mutations that confer growth advantage. Recent studies highlight a substantial heritable component to CH, implicating germline mutations in DNA damage repair (DDR) genes. These genes are essential for maintaining genomic integrity and pathogenic variants in key DDR genes are well-established genetic underpinnings of several hereditary cancer syndromes. This review synthesizes current data linking germline DDR mutations – including *ATM*, *CHEK2*, *TP53*, *PPM1D*, *BRCA1/2*, and *PARP1 –* to CH and the development of myeloid malignancies.

**Recent Findings:**

Emerging evidence suggests that germline perturbations in DDR pathway contribute to CH, though mechanisms remain incompletely defined. Large scale genome-wide association studies (GWAS) have identified strong associations between *ATM* and *CHEK2* variants and CH. Assessing prevalence and CH risk in individuals with germline *TP53* variants presents unique challenges, as distinguishing between somatic and constitutional lesions is often complex and requires careful tissue evaluation. The link between germline *BRCA1/2* and CH remains inconclusive, confounded by concurrent diagnosis of solid malignancy and prior exposure to chemoradiation therapy in studied patient populations. Although germline mutations in *PPM1D* and *PARP1* are rare, a potential germline predisposition to CH cannot be excluded.

**Summary:**

The totality of current evidence suggests that germline DDR pathway mutations not only predispose to well-established solid malignancy syndromes but also to CH, which independently increases the risk of hematologic malignancies. Recognizing germline contributions to CH has broad implications for risk assessment, surveillance strategies, and development of preventive strategies in myeloid neoplasia.

## Introduction

Clonal hematopoiesis (CH) arises when hematopoietic stem cells acquire somatic mutations that confer a proliferative advantage, leading to the expansion of mutant clones. Initially associated with the aging process, CH is understood to be influenced by inherited genetic factors. Recent genome-wide association studies of CH carriers have identified multiple genetic loci where germline variations influence predisposition to CH [1, 2]. Specifically, germline variations in DNA damage repair (DDR) genes have emerged as key determinants of CH risk later in life.

The DDR genes collectively preserve genomic integrity by activating checkpoints, coordinating repair mechanisms, and inducing cellular apoptosis or senescence in the event of unsuccessful repair. Proper functioning of DDR genes is crucial for hematopoietic stem cells as they experience continual genotoxic stress from DNA replication, environmental exposure, and cancer therapies such as radiation and cytotoxic chemotherapy. Therefore, it is unsurprising that germline variations in DDR genes may create a permissive environment for the acquisition and survival of somatic mutations that underlie CH. Mutations in DDR genes are also frequently observed in various hematologic disorders. In this review, we examine the impact of germline DDR gene variants on CH and myeloid neoplasms.

### Germline Predisposition to Clonal Hematopoiesis

Classic hereditary hematologic disorders are often characterized by pathogenic germline mutations that directly contribute to the syndromic pathophysiology and follow Mendelian patterns of inheritance. The age at presentation of such disorders varies with patients developing symptoms in childhood (e.g. Noonan syndrome, Fanconi Anemia, Tatton-Brown-Rahman), or in adulthood (e.g. *ANKRD26*, *ETV6*, *RUNX1*, *CEBPa*) [3, 4]. Widespread use of next generation sequencing (NGS) used primarily to detect somatic changes that occur with malignancy and often portend risk [5–7] or response to therapy [8–10] has uncovered genetic changes that occur at 45–55% allele burden (thus implying possible germline variants). Most commonly, this occurs among patients with previously deemed idiopathic hematologic malignancies. The implication of these germline variants has been further complicated by variable penetrance patterns and complex interactions with other genes. For example, while germline *DDX41* mutations are inherited in an autosomal dominant fashion, only 25–50% of *DDX41* carriers develop *DDX41*-associated acute myeloid leukemia at a median age of onset of 71 years [11, 12]. And while many germline variants are sufficient to lead to a disease phenotype, some exert their influence and disease predisposition in how they impact the acquisition of somatic changes.

Conceptually, this was first commonly recognized in familial clusters of myeloproliferative neoplasms (MPN) patients with somatic *JAK2-V617F* variants [13, 14]. Importantly, a shared germline haplotype GGCC “46/1” predispose a 3–4 fold risk of developing a somatic *JAK2 -V617F* variant which is causative of MPN [14–16]. Subsequently, genome-wide association studies (GWAS) of patients with *JAK2 -V617F*-CH and MPN revealed additional associations with germline variants in *TERT*, *SH2B2*, *TET2*, *ATM*, *CHEK2*, *TINT* and *GFI1B* genes [17]. Of these, different non-coding *TERT* variants have been shown to confer variable risks for MPN development, with intron 2 variants having significant association with more aggressive MPN phenotype and intron 3 variants associated with more indolent course of asymptomatic CH [17, 18].

Mosaic loss of the Y chromosome (mLOY), the most common somatic genetic lesion in men is associated with clear germline predisposition [18–20]. Initially mLOY was found to be associated with common SNP near the 5’ end of *TCL1A* gene (rs2887399), and subsequent large scale GWAS studies aiming to identify functional transcripts that contribute to mLOY identified eight additional contributing genes, many of which are involved in cell cycle progression and DNA damage response [19–21].

### Mutations in DNA Damage Repair Genes and Predisposition to Clonal Hematopoiesis

**Fig. 1:**
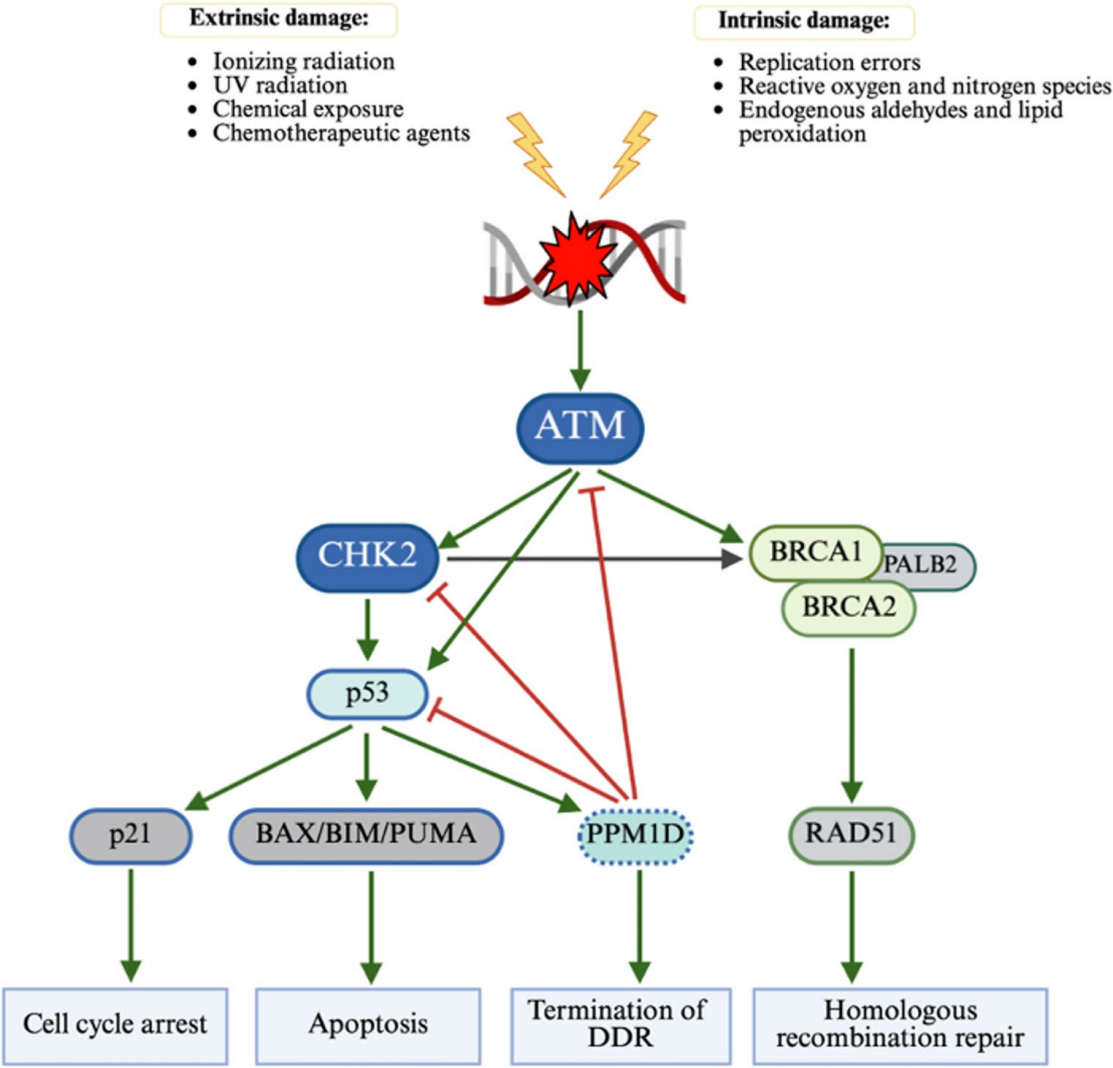
**ATM signalling in DNA damage repair (DDR response)** ATM is the nexus of DNA damage response. After extrinsic DNA damage, activated ATM phosphorylates several downstream targets including CHK2 and BRCA1 (not visualized). CHK2 is essential to the propagation of DDR signal to the downstream effectors including but not limited to p53 and BRCA1. Activated p53 engages p21 to trigger cell cycle arrest to allow for DNA repair, or BAX/BIM/PUMA cascade to initiate apoptosis. p53 also recruits a negative regulator PPM1D which functions to terminate DDR response once DNA repair is complete. Following activation by CHK2, BRCA1 recruits BRCA via PALB2 which then activates RAD51 for homologous recombination repair

Carcinogenesis is marked by an accumulation of mutations that provide a clonal advantage and ultimately genomic instability. This is a feed-forward process with increasing genomic instability leading to oncogene activation or loss of a tumor suppressor function [22, 23]. Genes involved in DNA Damage Repair (DDR) pathways are essential to monitoring and coordinating DNA repair, safeguarding the cells from propagating tumorigenic mutations during cellular division. Complex signaling cascades involved in DDR are mediated by checkpoint kinases. Mutations within DDR pathways, including *ATM*,* CHEK2*, *BRCA1/2*, *PALPB2* and *TP53* have been associated with development of a broad spectrum of tumors [24, 25]. While somatic mutations in DDR genes contribute to neoplastogenesis, particularly in selection of resistant cells after therapy, the germline influence of germline variants in DDR have impact across tumor types (Fig. 1).

Sustained efforts to characterize and understand factors contributing to CH have revealed a link between germline mutations in DNA damage repair genes and the development of CH. A large scale genome-wide association study (GWAS) on 184,121 people of European ancestry compared 10,203 individuals with CH to 173,918 without CH and identified 7 independent genome wide loci, including genes in DDR pathway, associated with risk of development of CH [1]. Here we discuss the important established links between germline DDR mutations and CH (Table 1).

**Table 1:**
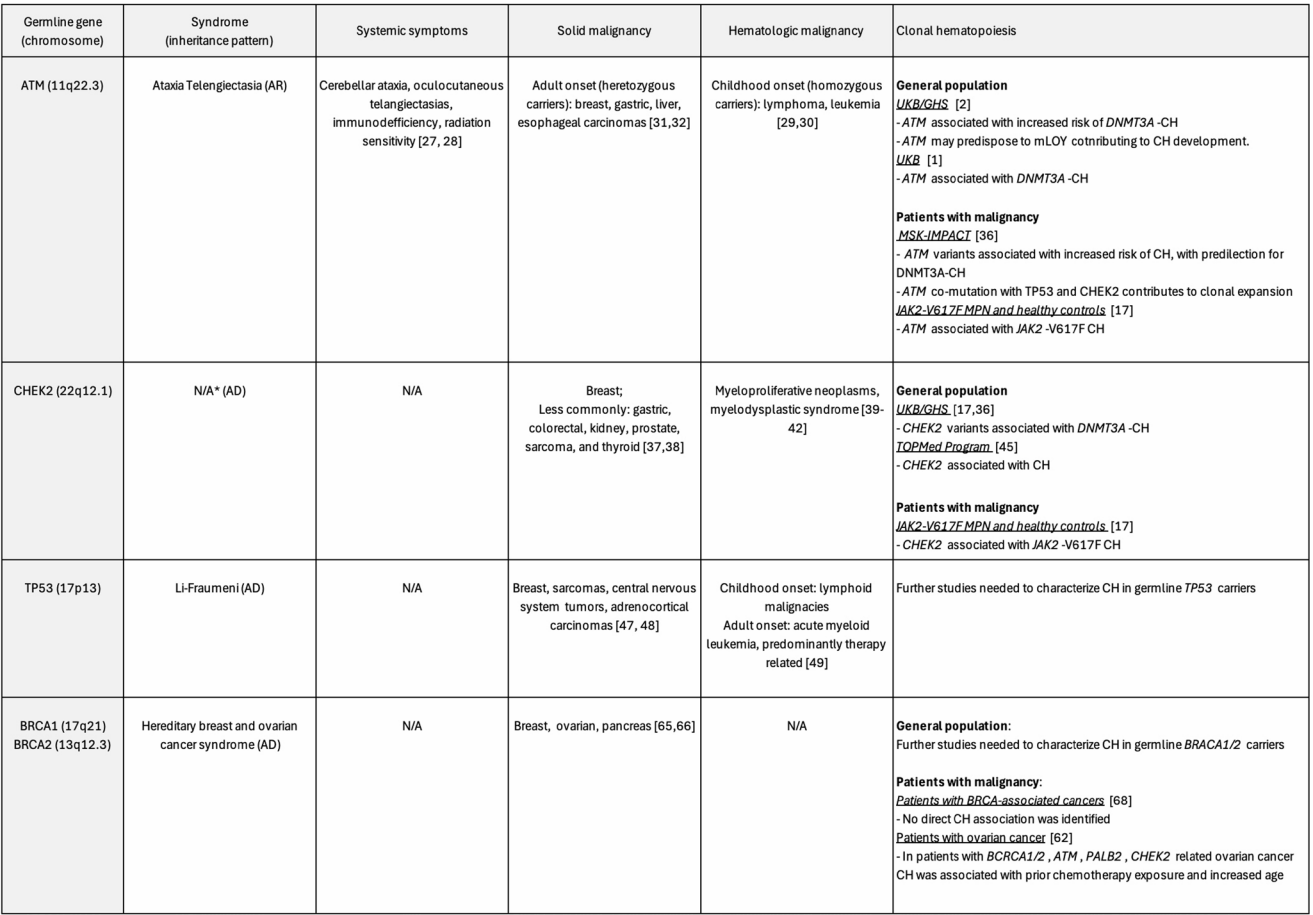
**Summary of Germline DDR mutations associated with malignancies and impact on development of clonal hematopoiesis**

### Germline ATM Mutations

Ataxia telangiectasia mutated (ATM) is a principal DNA damage checkpoint serine/threonine kinase central to DNA double-strand break repair, particularly during G1-S and G2-M checkpoints of the cell cycle [26, 27]. The most well recognized condition associated with germline *ATM* mutations is ataxia-telangiectasia, an autosomal recessive neurodegenerative disorder characterized by progressive cerebellar ataxia, cutaneous telangiectasias, immunodeficiency, increased risk of lymphoid malignancies, and radiosensitivity [27, 28]. The onset of malignancies associated with *ATM* deficiency appear to stem from the mutation burden of *ATM* mutations. Homozygous *ATM* mutations uniquely predispose to development of childhood lymphoid malignancies due to *ATM* dependent repair of DNA double-stranded breaks during V(D)J recombination and class switch in B and T lymphocyte development [29, 30]. By contrast, heterozygous pathogenic *ATM* variants are associated with increased risks of malignancy in adulthood [31, 32].

Although *ATM* mutations drive hematologic malignancies, *ATM* mutations occur in less than 1% patients with AML and MDS [33]. The mechanisms via which *ATM* contributes to myeloid leukemogenesis remain poorly defined but are thought to stem from impaired cell cycle checkpoints ultimately leading to accumulation of genetic lesions and chromosomal instability, consistent with ATM’s critical role in DDR [20, 34].

While *ATM* mutations are not considered to be the founding events of myeloid leukemogenesis they have been associated with impaired self-renewal of hematopoietic stem and progenitor cells (HSPCs) and have been implicated in providing selective advantage to mutated HSPC [35]. Consequently, expansion of such mutated HSPCs could contribute to the development of clonal hematopoiesis. Recent genome-wide and exome-wide association analyses of healthy volunteer samples from United Kingdom (UK) Biobank and Geisinger Health System (GHS) identified rare germline *ATM* variants that were significantly associated with CH [2]. Separate analysis of UKB data also implicated *ATM* as gene associated with CH [1]. Additionally, germline *ATM* has been linked to increased risk of *JAK2* V167F-CH in healthy population and to *JAK2* V617F mutated MPN in previously diagnosed patients. This risk was independent from that of previously known predisposition emerging form 46/1 *JAK2* haplotype [17].

Prospective tumor-blood paired sequencing of 46,906 patients from MSK-IMACT initiative, primarily including patients with advanced Stage III-IV cancers, showed that germline *ATM* variants were specifically associated with clonal hematopoiesis. Additionally, there was a strong association between germline *ATM* and somatic *ATM*-CH, with similar findings observed in patients with germline *TP53* mutations, suggesting that bi-allelic inactivation may provide growth advantage, select for larger clonal size and thus contribute to clonal expansion [36]. Separately, single-nucleotide polymorphism associated with ATM has also been identified as one of causative genes and predisposition to mLOY which in its turn predisposes to further clonal hematopoiesis [19, 20].

### Germline CHEK2 Mutations

*CHEK2* gene encodes for the checkpoint kinase 2 (CHK2), an effector serine/threonine kinase with critical role in cell cycle regulation, functioning as a tumor suppressor [37]. *CHEK2* variants were first described among families with Li- Fraumeni-like syndrome without germline *TP53* mutation [38]. Truncating variant c.1100delC is the most studied *CHEK2* variant and carries a strong association with increased risk of early-onset breast cancer (37% cumulative risk). The association with other cancers such as gastric, colorectal, kidney, prostate, sarcoma and thyroid is less well established [37, 38].

Mutations in germline *CHEK2* are seen in 2–3% of general population with the incidence higher in certain populations (p.S428F in Ashkenazi Jewish population and p.I200T in Eastern European populations). The majority of *CHEK2* mutations are germline with somatic variants being quite rare [39]. Mutations in *CHEK2* were previously thought to be uncommon in hematologic malignancies, with more recent studies suggesting that the true incidence may be as high as 15%, with certain populations having higher frequencies [39–42]. In large public AML/MDS databases such as BEAT AML, Leucegene, AML PMP, *CHEK2* mutations were found in 1.2% of AML patients [41]. The incidence was higher in patients suspected to have hereditary hematologic malignancy syndrome, where 3.6% were found to have *CHEK2* variants, with majority having p.I157T (53%), followed by pT367fs (c.100delC, 11%) variants [41].

Surprisingly, whole exome sequencing on paired leukemia and skin biopsy samples revealed 13.6% leukemia patients had germline variants in *CHEK2*. These findings were replicated in a small study of post-remission bone marrow samples of 47 patients with AML who were not suspected to have hereditary predisposition [43, 44]. Among 205 germline variants detected across 110 genes, only 11 were classified as pathogenic or likely pathogenic, with most localizing to DNA damage response genes, including *ATM*, *CHEK2*, *FANCA*, *FANCM* [43].

Germline *CHEK2* mutations have been linked to development of CH in multiple large scale GWAS [1, 2, 17, 36]. In analysis of UKB and GHS data, germline *CHEK2* truncating variants were strongly associated with *DNMT3A*-CH [2]. A separate analysis of a smaller dataset from the UK Biobank revealed that the c.100delC germline *CHEK2* variant confers a markedly increased risk of *DNMT3A*-CH with the effect size 4 times larger than that of the other common risk alleles such as *ATM*, *TERT*, or *SCM4* [1]. In gene-based association tests for aggregation of rare germline variants among participants of the National Heart, Lung, and Blood Institute Trans-omics for Precision Medicine (TOPMed) cohort while no genes reached exome-wide significance, *CHEK2* was found to be the top associated gene [45]. Similar to germline *ATM*, *CHEK2* has been closely associated with *JAK2-V617F-*CH and MPN [17].

Collectively, these findings underscore the importance of CHK2 in preserving genomic stability in hematopoietic stem cells and provide a mechanistic link between inherited DNA repair defects and the development of clonal hematopoiesis with high risk for subsequent myeloid malignancy.

### Germline Mutations in TP53

*TP53* gene, commonly referred to as the “Guardian of the genome” encodes p53 tumor suppressor protein. P53 is essential to regulating cell cycle arrest, apoptosis, and cellular senescence in response to in response to cellular stress signals, particularly DNA damage [46]. Li-Fraumeni syndrome – a rare autosomal dominant syndrome defined by germline mutations in *TP53* – confers a strong predisposition to broad spectrum of malignancies including breast cancer, soft tissue sarcomas, osteosarcomas, brain tumors, adrenocortical carcinomas, leukemia, and lymphoma [47, 48]. Hematologic malignancies, lymphoid as well as myeloid, in Li-Fraumeni syndrome are less common, affecting less than 10% of carriers [49–52].

In a recent study of Li-Fraumeni syndrome associated hematologic malignancies, age at the time of diagnosis ranged significantly, with B-ALL developing at a median age of 12 years old, non-Hodgkin lymphoma at 39 years old, and MDS/AML at 33 years old [49]. The patients diagnosed with hematologic malignancies without prior chemotherapy exposure were younger (14 years) and had predominantly lymphoid phenotype (81% ALL), compared to those with prior chemotherapy exposure who were older (31 years) and had myeloid phenotype (79% MDS/AML). Nearly half of those diagnosed with secondary myeloid malignancies had multi-hit *TP53* loss.

Li-Fraumeni syndrome is phenotypically and genomically diverse, ranging from individuals with personal and family history of multiple neoplasms fulfilling clinical criteria despite the absence of a germline *TP53* mutation, to asymptomatic germline *TP53* carriers lacking any personal or family history of malignancy [47].

The diagnostic challenge is further compounded by the risk of misclassifying high-allele frequency *TP53* mutations identified by sequencing of the peripheral blood samples as germline. Several studies of paired blood-tumor samples highlight the need for more nuanced diagnostic approach than peripheral blood testing as corresponding tumor samples may not contain any *TP53* alterations and thus are not part of Li-Fraumeni syndrome spectrum [53, 54].

The analysis of paired blood-tumor samples from MSK-IMPACT revealed that of 17,992 patients with solid malignancies only 50 (0.28%) individuals had *TP53* mutations, with only 38 (0.19%) confirmed to be of germline origin. Yet still, confirmed germline *TP53* does not directly correlate with full Li-Fraumeni phenotype. Loss of heterozygosity (LOH) is the critical molecular event that leads to tumorigenesis that manifests as Li-Fraumeni syndrome. While 96% of the patients with true Li-Fraumeni tumor phenotype having concurrent LOH of wild-type *TP53* and germline *TP53* mutation, only 46% of non-Li-Fraumeni tumor phenotype patients have such double-hit *TP53* loss [55].

Furthermore, the timing of sample collection in suspected germline *TP53* carriers plays a significant role in result interpretation, as somatic *TP53* is one of the most common CH lesions [45, 56–58], In the paired blood-tumor analysis from MSK-IMPACT the samples obtained from older patients, or following chemotherapy administration were found to have higher rate of somatic *TP53* mutations most likely representing chemotherapy-included CH rather than germline *TP53* variants [53, 55].

In recently published analysis of 140,597 participants without hematologic neoplasms from BioBank Japan, *TP53*-CH was associated with poor overall survival (HR 1.42) [59]. Not unexpectedly, individuals with *TP53*-CH were found to have increased mortality from myeloid and lymphoid hematologic malignancies compared to non-carriers. This adverse prognostic impact was evident even among those without prior malignancies, indicating that the risks is not solely attributable to secondary myeloid neoplasms [59].

Differentiating between somatic *TP53*-CH and Li-Fraumeni syndrome poses a clinical challenge due to significant heterogeneity in the phenotype [47]. To date CH in patients with *TP53* mutations in setting of Li-Fraumeni syndrome remains poorly characterized.

### Germline PPM1D Mutations

PPM1D (Protein Phosphatase, Mg2+/Mn2 + Dependent 1D) is a serine/threonine phosphatase that functions to attenuate DDR response by dephosphorylating substrates in the p53 and checkpoint pathway, including p53 itself, ATM, and CHK1/2 [60]. Most pathologic variants in *PPM1D* gene located on chromosome 17q are somatic truncating mutations in exon 6 that precludes proteasomal degradation while maintaining proteolytic activity [61].

*PPM1D* mutations are rare in general population (0.13–0.16%) and are primarily seen in a setting of clonal hematopoiesis in patients with prior chemotherapy or radiation exposure (2.5–13%) [18, 62]. While somatic mutations in *PPM1D*-CH are well recognized, germline mutations in *PPM1D* are rare and primarily described in setting of developmental delay and various congenital abnormalities [63]. Although *PPM1D* mutations have been identified in patients with CH, particularly in patients with prior chemoradiotherapy exposure, GWAS of patients with CH and/or myeloid malignancies did not identify underlying germline *PPM1D* mutations [1, 2].

### `Germline BRCA1/2 Mutations

*BRCA1* and *BRCA2* are tumor suppressor genes within DDR pathway, critical for facilitating repair of double stranded DNA breaks via homologous recombination [64]. Germline mutations in *BRCA1* and *BRCA2* markedly increase the risk of several solid malignancies, most notably breast, ovarian, and pancreatic cancers. By age 70, the cumulative risks of breast cancers is estimated at 60% for *BRCA1* mutation carriers and 50% for *BRCA2* mutation carriers. The risk of ovarian cancer is substantially higher in *BRCA1* mutation carriers, approximately 50% by age 70, compared to 11% for *BRCA2* mutation carriers [65, 66].

It has been proposed that the underlying DDR defects in *BRCA1/2* mutated cancers increase the risk of development of CH. In a retrospective analysis of 24,849 patients with breast, ovarian, prostate and pancreatic cancers with DDR mutations (*BRCA1/2*,* ATM*,* PALB2*,* CHEK2*) Marshall et al. found that 14% patients had CH [67, 68]. Among all patients evaluated, the presence of CH correlated with increased age at the time of biopsy but following age adjustment only breast cancer was found to be associated with CH (OR 1.41). The most common types of CH in this cohort were *DNMT3A*-CH (37%), *PPM1D*-CH(37%), *TET2*-CH, (16%), *TP53*-CH (8.5%) and *ASXL1*-CH (8.6%). Among other cancer types, there was no association between CH and any specific DDR mutation. The authors propose that findings suggest that the risk of development of CH is influenced by underlying cancer biology and selective therapeutic pressures [68].

In a different study evaluating the incidence and risks for CH in patients with ovarian cancer Weber-Lassalle et al. found that out of 448 patients 17% were found to have at least 1 CH-associated mutation [62]. Patients with CH were significantly older at the time of testing and were more likely to have had platinum-based chemotherapy within 30 days of sample collection. Among the patients with platinum-based therapy exposure *PPM1D* and *TP53*-CH were the most common. No association between *BRCA1/2* mutation status (20.7% cohort) and development of CH was seen in this patient cohort.

## Germline PARP1 Mutations

PARP1 is a key nuclear enzyme involved in detection and repair of DNA single- and double-strand breaks by poly(ADP-ribosyl)ation. Impaired activity of PARP1 results in accumulation of DNA damage and thus increased susceptibility for malignant transformation [69]. Germline mutations in *PARP1* are uncommon and to this day have not been associated with well-defined syndromes.

In a GWAS of UK Biobank participants, *PARP1* variants were found to be associated with an overall increased risk of CH. Among these, the well-recognized G-allele (rs1126410) showed a subtype dependent effect, with negative association with *DNMT3A*‑ and positive with *TET2*‑mutant CH. The inverse relationship between rs1126410 – which is associated with reduced PARP1 catalytic activity - and *DNMT3A*-CH has led to the hypothesis that *DNMT3A*-CH may be particularly vulnerable to PARP1 inhibition, as supported by observed synergy between DNMT3A and PARP inhibitors [1, 70].

##  Conclusions

**Fig. 2:**
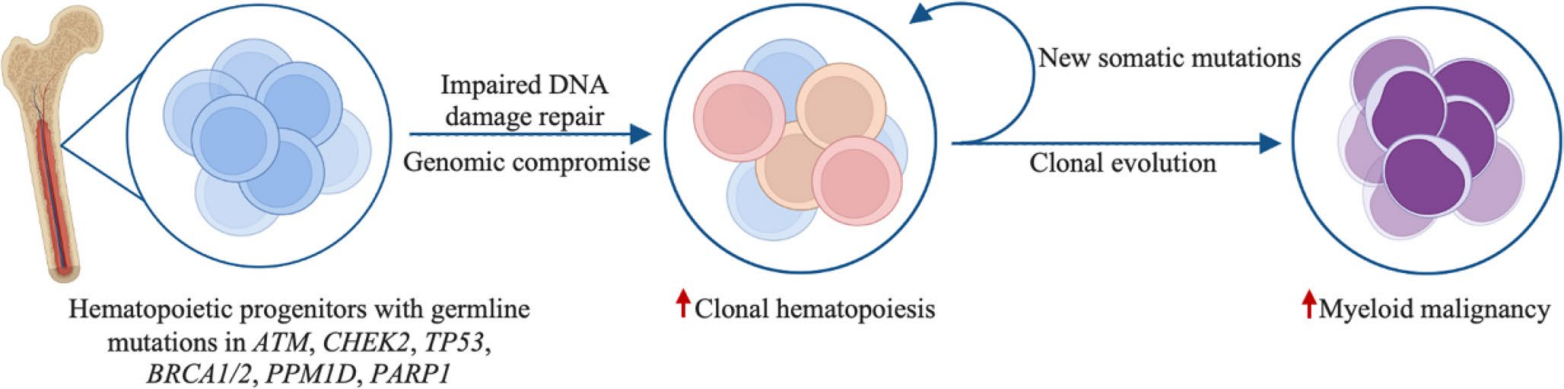
**Proposed model of germline DNA repair deficiency promoting clonal hematopoiesis and development of myeloid malignancy** Hematopoietic progenitors harboring germline mutations in DDR genes (*ATM*, *CHEK2*, *TP53*, *BRCA1/2*, *PPM1D*, *PARP1*) exhibit impaired DNA repair capacity, leading to genomic instability. The accumulation of un-repaired DNA lesions facilitates the emergence of clonal hematopoiesis, wherein progressive accumulation of somatic mutations drives clonal evolution and ultimate transformation towards myeloid malignancy

Germline mutations in DNA damage repair genes are implicated in nearly all tumor types. Recent large scale GWAS have recognized associations between these mutants and acquired CH. This begs the question as to how DNA damage repair mutations may increase the risk of acquiring CH, or provide a cellular context that allows rapid expansion of CH and development of malignancy. It is conceptually logical that germline mutations in DNA damage repair genes heighten cellular vulnerability to external cytotoxic stressors (e.g. chemotherapy or radiation) further suggesting that CH is not merely a byproduct of aging and environmental exposures, but can be a manifestation of compromised genomic integrity (Fig. 2). Still, the precise cooperative biologic activity between CH genes engaged in key cellular functions and these myriad germline DNA damage repair gene mutations in carcinogenesis remains unclear.

## Key References

- A. J. Silver, A. G. Bick, and M. R. Savona, “Germline risk of clonal haematopoiesis,” Nat Rev Genet, vol. 22, no. 9, pp. 603–617, Sep 2021, doi: 10.1038/s41576-021-00356-6.S. P. Kar et al., “Genome-wide analyses of 200,453 individuals yield new insights into the causes and consequences of clonal hematopoiesis,” *Nat Genet*, vol. 54, no. 8, pp. 1155–1166, Aug 2022, doi: 10.1038/s41588-022-01121-z.
  - **○ **This review article provides a comprehensive overview of clonal hematopoiesis, with a particular emphasis on heritable risk factors.
- M. D. Kessler et al., “Common and rare variant associations with clonal haematopoiesis phenotypes,” *Nature*, vol. 612, no. 7939, pp. 301–309, Dec 2022, doi: 10.1038/s41586-022-05448-9.
  - **○ **This original article presents findings from genome-wide association studies providing comprehensive overview of germline variants that contribute to predisposition to clonal hematopoiesis.
- S. Franch-Exposito et al., “Associations Between Cancer Predisposition Mutations and Clonal Hematopoiesis in Patients With Solid Tumors,” *JCO Precis Oncol*, vol. 7, p. e2300070, Aug 2023, doi: 10.1200/PO.23.00070.
  - **○ **This original article presents findings from prospective tumor-blood paired sequencing, demonstating association between germline variants in established malignancy predisposition genes and clonal hematopoiesis.
- R. J. Stubbins, S. Korotev, and L. A. Godley, “Germline CHEK2 and ATM Variants in Myeloid and Other Hematopoietic Malignancies,” *Curr Hematol Malig Rep*, vol. 17, no. 4, pp. 94–104, Aug 2022, doi: 10.1007/s11899-022-00663-7.
  - **○ **This review article provides a comprehensive overview of the role of deleterious germline mutations in *CHEK2* and *ATM* genes in the pathogenesis of hematologic malignancies.
- C. H. Marshall et al., “Germline DNA Repair Gene Mutations and Clonal Hematopoiesis (CH) in 24,849 Patients with BRCA-Associated Cancers,” *Cancers (Basel)*, vol. 17, no. 9, Apr 25 2025, doi: 10.3390/cancers17091432.
  - **○** This origimal article presents findings from a retrospective analysis of breast, ovarian, prostate, and pancreatic tumor samples from Tempus database evaluating the risk of clonal hematopoiesis in patients with germline mutations in homologous recombination repair genes.

## Data Availability

No datasets were generated or analysed during the current study.
